# GC-MS profiling of volatile metabolites produced by *Klebsiella pneumoniae*

**DOI:** 10.3389/fmolb.2022.1019290

**Published:** 2022-10-18

**Authors:** Wojciech Filipiak, Karolina Żuchowska, Marta Marszałek, Dagmara Depka, Tomasz Bogiel, Natalia Warmuzińska, Barbara Bojko

**Affiliations:** ^1^ Department of Pharmacodynamics and Molecular Pharmacology, Faculty of Pharmacy, Collegium Medicum in Bydgoszcz, Nicolaus Copernicus University in Toruń, Bydgoszcz, Poland; ^2^ Department of Microbiology, Faculty of Pharmacy, Collegium Medicum in Bydgoszcz, Nicolaus Copernicus University in Toruń, Bydgoszcz, Poland

**Keywords:** volatile metabolites, bacteria markers, gas chromatography -mass spectrometry (GC-MS), *Klebsiella pneumoniae*, ventilator-associated pneumonia (VAP), breath markers, headspace analysis, volatile organic compounds (VOC)

## Abstract

Currently used methods for diagnosing ventilator-associated pneumonia (VAP) are complex, time-consuming and require invasive procedures while empirical antibacterial therapy applies broad spectrum antibiotics that may promote antimicrobial resistance. Hence, novel and fast methods based on alternative markers are needed for VAP detection and differentiation of causative pathogens. Pathogenic bacteria produce a broad range of volatile organic compounds (VOCs), some of which may potentially serve as biomarkers for microorganism identification. Additionally, monitoring of dynamically changing VOCs concentration profiles may indicate emerging pneumonia and allow timely implementation of appropriate antimicrobial treatment. This study substantially extends the knowledge on bacterial metabolites providing the unambiguous identification of volatile metabolites produced by carbapenem-resistant and susceptible strains of *Klebsiella pneumoniae* (confirmed with pure standards in addition to mass spectra match) but also revealing their temporary concentration profiles (along the course of pathogen proliferation) and dependence on the addition of antibiotic (imipenem) to bacteria. Furthermore, the clinical strains of *K. pneumoniae* isolated from bronchoalveolar lavage specimens collected from mechanically ventilated patients were investigated to reveal, whether bacterial metabolites observed in model experiments with reference strains could be relevant for wild pathogens as well. In all experiments, the headspace samples from bacteria cultures were collected on multibed sorption tubes and analyzed by GC-MS. Sampling was done under strictly controlled conditions at seven time points (up to 24 h after bacteria inoculation) to follow the dynamic changes in VOC concentrations, revealing three profiles: release proportional to bacteria load, temporary maximum and uptake. Altogether 32 VOCs were released by susceptible and 25 VOCs by resistant strain, amongst which 2-pentanone, 2-heptanone, and 2-nonanone were significantly higher for carbapenem-resistant KPN. Considerably more metabolites (*n* = 64) were produced by clinical isolates and in higher diversity compared to reference KPN strains.

## Introduction

Early detection of infectious disease, with an identification of a causative pathogen to the species level, is always of the greatest clinical importance. It is also one of the main tasks of a clinical microbiology laboratory. The main reason is that bacteria belonging to different species present diverse patterns of natural/intrinsic resistance to antimicrobials. Therefore, the empiric treatment scheme is the most effective when tailored with the highest precision to the most probable etiological factor. Knowing the causative agent of the infection without the necessity to cultivate it, gives the advantage to administer more precise antimicrobial therapy with a higher possibility of microorganism eradication.

The recent pandemic of severe acute respiratory syndrome coronavirus 2 (SARS-CoV-2) has resulted in a big number of patients requiring hospitalization in the intensive care unit (ICU), and the use of invasive mechanical ventilation (MV) (Grasselli et al., 2020; Lima et al., 2020; Razazi et al., 2020; Giacobbe et al., 2021; Moretti et al., 2021). MV often leads to multiple complications in ICU patients with ventilator-associated pneumonia (VAP), being the most common (Vincent et al., 2020). Several studies have reported the prevalence of VAP in MV patients with COVID-19 ranging from 48 to 79% (Luyt et al., 2020; Razazi et al., 2020; Blonz et al., 2021; Maes et al., 2021; Rouzé et al., 2021). The consequence of VAP on patient outcome was summarized in the comprehensive study of Koulenti et al., enrolling nearly 2500 patients from 27 Intensive Care Units (ICUs) across Europe: VAP was directly related to 19.6% and contributes to 43.9% of deaths at ICU, and in patients who survived it prolongs ICU stay for another 12 days, mechanical ventilation for 10 days, and increases the incidence of bacteremia by 14.6% (Koulenti et al., 2017). Given that a Clinical Pulmonary Infection Score seems to be inadequate as a “Stand Alone” approach for VAP diagnosis (3/6 CPIS criteria are present in 78%, but 6/6 CPIS criteria are present in 0.2% of VAP patients) (Koulenti et al., 2017) additional local and official criteria (such as European Centre for Disease Prevention and Control, ECDC) need to be involved in recognition of pneumonia (Module, 2019). Noticeably, the microbiological testing of an underlying pathogen requires invasive sampling of bronchoalveolar lavage (BAL) specimens and results are known after 2 days, during which patients receive an empirical therapy comprising broad-spectrum antibiotics, mainly carbapenems. Although timely antibiotic treatment is crucial to improving patient outcomes (Xie et al., 2019), the overuse of antibiotics results in the development of antimicrobial resistance (Martin-Loeches et al., 2018). According to ECDC Antimicrobial Resistance Report, in 2019 more than half of *Escherichia coli* isolates and more than a third of *Klebsiella pneumoniae* (KPN) isolates were resistant to at least one antimicrobial group under surveillance (Report, 2020). Antimicrobial resistance among *K. pneumoniae* increases due to the ability to acquire resistance mechanisms e.g. antibiotics hydrolyzing enzymes such as carbapenemases (*Klebsiella Pneumoniae* Carbapenemase – KPC, New Delhi metallo-beta-lactamase – NDM) (Galani et al., 2021). A natural step towards de-escalation of antimicrobial resistance seems to be the implementation of a therapy targeted to VAP causative pathogen. However, a fast and non-invasive method for the detection of VAP with simultaneous identification of the causative pathogen is needed to achieve this ambitious aim.

In the last years it has been confirmed, that microorganisms, including pathogenic bacteria, produce a broad range of Volatile Organic Compounds (VOCs) (Elmassry and Piechulla, 2020). Some of them may be considered unique to the pathogen and potentially serve as biomarkers for bacteria differentiation. Analysis of dynamically changing concentration profiles of bacteria-specific VOCs in the exhaled breath of MV patients might enable non-invasive detection of emerging pneumonia allowing fast implementation of appropriate antibiotic treatment and monitoring of the course of infection. For this purpose, the unambiguous identification of bacteria-derived metabolites is of utmost importance and requires both, a model study with reference strains and its verification with clinical isolates.

This study aims to characterize the kinetics of metabolites release and uptake by carbapenem-susceptible and -resistant strains of *K. pneumoniae*. Additionally, the effect of imipenem addition to the bacteria culture on VOCs production was investigated. It was also studied whether VOCs secreted from reference strains were produced by clinical strains isolated from VAP patients as well. In all experiments, the headspace samples from bacteria cultures were collected and preconcentrated on multibed sorption tubes and immediately analyzed on GC-MS. Culturing and sampling were done under strictly controlled ventilation conditions at several time points to follow the dynamic changes in temporal VOCs concentration profiles.

## Materials and methods

### Bacteria cultivation

Carbapenem susceptible (ref. No. 681) and carbapenem resistant (ref. No. 103517) strains of *K. pneumoniae* from German Collection of Microorganisms and Cell Cultures GmbH of Leibniz Institute (Leibniz Institut DSMZ - Deutsche Sammlung von Mikroorganismen und Zellkulturen GmbH) were investigated. Altogether six biological replicates of the carbapenem-susceptible strain and nine biological replicates of carbapenem-resistant strain were measured in this study (one technical replicate was used in all cases). Before experiments, strains were stored in Tryptic Soy Broth (TSB, Becton Dickinson, Franklin Lakes, NJ, United States) with 15% glycerol at a temperature of −80°C.

The clinical strains included in this study were derived from bronchoalveolar lavage samples collected from 19 patients suffering from ventilator-associated pneumonia, hospitalized in the Anesthesiology and Intensive Care Unit of the 10th Military Research Hospital and Polyclinic in Bydgoszcz, Poland. Initially, they were plated quantitatively on a set of media plates, according to standard microbiological procedures. The cultures were kept overnight at 37°C with the following qualitative read-out. The strains that reach a certain titer were identified and subjected to antimicrobial susceptibility testing and detection of the most important antimicrobial resistance mechanisms. At the next step the strains were re-cultured on non-selective media and stored at −80°C in a Brain Heart Infusion containing 10% glycerol for further testing.

All samples (i.e. reference strains and clinical isolates) were analyzed according to the same protocol after preculturing them on McConkey Agar (Becton Dickinson, Franklin Lakes, NJ, United States) at 37°C for 24 h. Bacteria were grown in a 3D culture suspended in a tryptic soy broth (TSB) medium with a stirring rate of 80 rpm. Plating for colony forming unit (CFU) counts has been performed on Difco TM MacConkey Agar plates. Bacteria identification and antimicrobial susceptibility tests (AST) were performed with Phoenix BD™ M50 NMIC/ID-402 panels (Becton Dickinson, Franklin Lakes, NJ, United States). The results of AST were interpreted according to European Committee on Antimicrobial Susceptibility Testing recommendation v 12.0 (EUCAST, 2022).

### Headspace sampling

**FIGURE 1:**
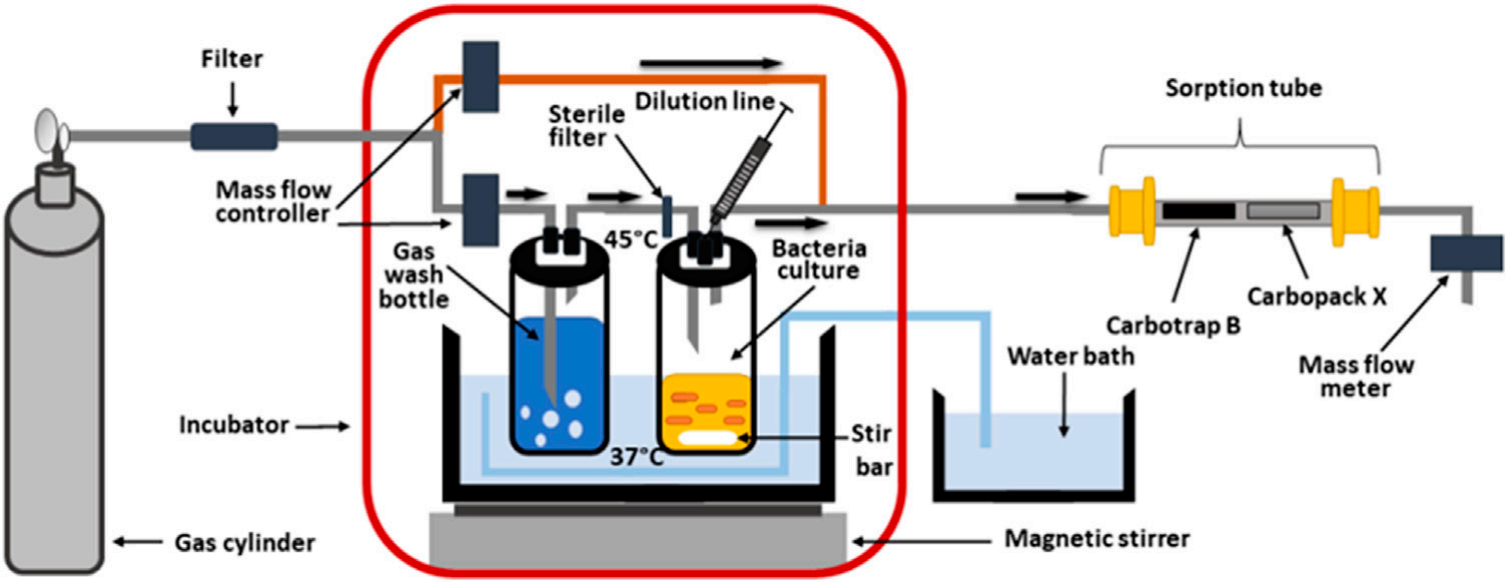
In-house-made system for cultivation of bacteria with simultaneous preconcentration of headspace gas on sorption tube.

An in-house-made system adapted from our previous study (Filipiak et al., 2012) and adjusted to sampling of headspace gas from bacteria culture was used (Figure 1). Glass bottles containing 100 ml of bacteria suspension in TSB were kept at 37°C within a water bath which was additionally placed inside an incubator at 45°C. Synthetic air of the purity 6.0 enriched with 5% CO_2_ (Air Products, Belgium) was additionally purified with Supelcarb filter (Merck KGaA, Darmstadt, Germany) and the flow was split into two lines: (A) low-flow (5 ml/min) passing the water purge and bacteria culture and (B) high-flow (40 ml/min) to dilute and decrease the humidity of bacterial headspace. All flows were precisely controlled with Mass Flow Controllers with an option of automatic flow compensation (Vögtlin Red-Y smart series, NewTech, Gliwice, Poland) and also flows through the sorption tubes were additionally checked during the sampling at the outlet from the system on Mass Flow Meter (Vögtlin Red-Y Compact, NewTech, Gliwice, Poland). Sorption tubes were filled with 140 mg of Carbotrap B (20/40 mesh) and 330 mg of Carbotrap X (60/80 mesh) ensuring the coverage of a wide range of VOCs with simultaneous low water uptake (additionally reduced by dilution line). The potential changes in the composition of a tryptic soy broth have been thoroughly investigated in our previous study, where the experimental protocol was optimized to ensure only minimum VOCs emission from TSB medium to the headspace, hence a very stable background over the course of the experiment, as described in Filipiak et al., 2013 (Filipiak et al., 2013). The volumes of 200 ml of headspace gas for GC-MS analysis were taken at 0 h (T0), 2 h 40min (T1), 4 h 10min (T2), 5 h 40min (T3), 7 h 10min (T4), 8 h 40min (T5) and 24 h(T6) after inoculation of bacteria to a pure TSB medium. After each headspace sampling, 300 µl of bacterial suspension was collected to provide an assessment of bacterial growth and its quantification (CFU/ml). For every taken suspension, three dilutions in TSB in the range from 10^−4^ to 10^−8^ were performed, then 100 µl were plated on MacConkey Agar plates and left at 37°C for 24 h. Then the counts of colony forming units (CFU) were performed.

In further experiments, the influence of imipenem on sensitive and resistant *K. pneumoniae* reference strains was investigated. For this purpose imipenem solution in PBS at a concentration of 25 mg/100 ml was prepared and 0.5 ml of it was added to each of the four bottles with bacterial cultures, at T2 immediately after collection of headspace gas and bacteria suspension.

### Thermal desorption Gas Chromatography mass spectrometry (TD-GC-MS)

A TD-30R autosampler (Shimadzu, Shim-Pol, Warsaw, Poland) was used for the thermal desorption of entrapped bacterial metabolites into the GC-MS system. As a carrier gas for thermal desorption, Helium 6.0 purified on an HP-2 Heated Helium Purifier (ViCi Valco Instruments, Houston, TX, United States) and an additional double set of Carrier Gas Purifiers (Agilent, Santa Clara, CA, United States) was used with a flow of 60 ml/min. Desorption of samples took place at 320°C over 15 min. The desorbed gases were focused on a cold trap at −20°C filled with Carboxen. Injection into a Nexis 2030 Gas Chromatograph (Shimadzu, Shim-Pol, Warsaw, Poland) took place at 350°C over 2 min in split-less mode with a carrier gas flow of 0.82 ml/min (linear velocity 33.0 cm/s). Chromatographic separation was done on Rt-Q-Bond capillary column 30 m × 0.25 mm × 8 µm (Restek, Bellefonte, PA, United States) using the following temperature program: initial 60°C held for 2 min, then ramped 8°C/min to 110°C at 1 min, ramp 3°C/min to 120°C at 7 min, ramp 3°C/min to 155°C at 7 min, ramp 3°C/min to 225°C at 4 min, ramp 10°C/min to 300°C at 7 min. Data acquisition was done with a QP-2020-NX Mass Spectrometer (Shimadzu, Shim-Pol, Warsaw, Poland), working in SCAN mode (within 33–235 m/z range).

### Data processing and statistical analysis

Detected compounds were identified first by matching the acquired spectrum with the NIST 2017 Mass Spectra Library (Gaithersburg, MD, United States) and additionally confirmed by the retention time of a reference substance. All substances used for the identification of detected metabolites were purchased from Alchem (Alchem, Torun, Poland). Chromatographic peaks were initially integrated using a customized method in Shimadzu GCMS Postrun Analysis software and further, if necessary, manually corrected by an experienced GC-MS analyst. The statistical significance between VOC levels in bacterial suspensions (given as peak areas for respective chromatographic peaks) at different growth times and reference TSB medium was calculated in Statistica 13.3 PL software (TIBCO Software Inc., Tulsa, OK, United States) using the Kruskal-Wallis test, which is a nonparametric test to compare samples from two or more groups of independent observations, where *p*-values < 0.05 were considered to be significant. This test was selected because of its stability to outliers and no requirement for the groups to be normally distributed. For a narrower comparison of VOC abundance between only two groups of interest (at the same timepoint of an experiment) the non-parametric U-Mann-Whitney test was chosen with additional False Discovery Rate correction using the Statistica 13.3 software. For so selected significant VOCs, the peak area was further log-transformed and plotted using Statistica 13.3 PL and Metaboanalyst 5.0 online software (Author anonymous, 2021).

## Results

### Bacteria proliferation

**FIGURE 2:**
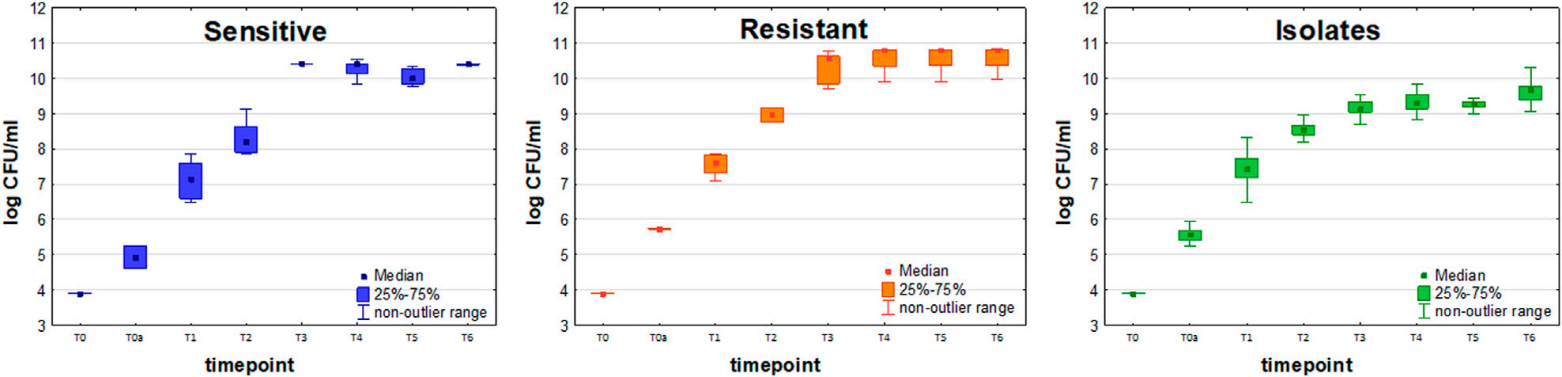
Growth curves of sensitive (blue boxes, *n* = 6), resistant (orange boxes, *n* = 9) and clinically isolated (green boxes, *n* = 23) strains of *Klebsiella pneumonia*. Colony Forming Units (CFU/ml) are plotted after logarithmic transformation in the function of incubation time.

Initial bacteria density (measured immediately after inoculation) ranged from 4 × 10^4^ CFU/ml to 1.8 × 10^5^ CFU/ml for the sensitive strain (*n* = 6) and from 5.4 × 10^5^ CFU/ml to 5.6 × 10^5^ CFU/ml for resistant strain (*n* = 9). Slightly lower values were observed for sensitive compared to resistant strain, with the mean values at the end of experiment 2.6 × 10^10^ CFU/ml at 4.7 × 10^10^ CFU/ml, respectively. A control measurement was performed at 24 h since inoculation to confirm that a steady state in bacteria growth was reached, after which the experiment was discontinued (Figure 2).

To compare the proliferation kinetics of both reference strains of *K. pneumoniae*, the non-parametric U-Mann-Whitney test was performed by Statistica 13.3 software. According to the results obtained, no statistically significant differences could be observed between the proliferation of resistant and sensitive strains at the same timepoints, except T2 (Table 1) after which the growth curves for both strains were coherent again. This indicates, that the dynamics of cell division and resulting bacteria load is similar, hence it is appropriate to directly compare the amounts of VOCs released from sensitive and resistant strains of *K. pneumoniae* when considering a particular timepoint.

**TABLE 1:** Statistical comparison of proliferation rate between susceptible and resistant strains of Klebsiella pneumonia.

Timepoint	Mean CFU/ml for susceptible strain	Standard deviation [CFU/ml] for susceptible strain	Mean CFU/ml for resistant starin	Standard deviation [CFU/ml] for resistant strain	*p*-value
T1	2,7333E+07	2,5876E+07	4,1111E+07	2,1707E+07	0.2880
T2	3,8500E+08	4,5121E+08	9,7444E+08	3,7813E+08	**0.0156**
T3	1,1333E+10	6,1824E+09	2,8333E+10	2,0309E+10	0.3139
T4	2,4333E+10	5,8784E+09	4,5111E+10	2,1957E+10	0.2139
T5	2,4833E+10	6,1486E+09	4,5556E+10	2,2006E+10	0.2143
T6	2,5500E+10	6,1033E+09	4,7111E+10	2,2728E+10	0.2109

*p*-values are given for the non-parametric Wilcoxon rank-sum test with FDR correction, whereby the threshold of *p* = 0.05 was chosen for significance.

Amongst 19 KPN clinical isolates collected from VAP patients, only two were imipenem-resistant, hence statistical comparison of VOCs profiles between sensitive and resistant clinical strains was impossible. Due to this reason, the isolates are referred only to as the sensitive reference strain in further data interpretation. The proliferation rate of clinical strains was lower than reference sensitive strains and statistically significant differences in bacteria load between these two sets were observed for T3, T4, T5, and T6 timepoints using a U-Mann-Whitney test.

### Time-dependent profiles of metabolites production of *K. pneumoniae*

Altogether 44 VOCs were observed at significantly different levels in the headspace of the sensitive KPN compared to the control medium for at least one timepoint in the course of the experiment, amongst which 32 VOCs were released and 12 VOCs were taken up by bacteria culture (Tables 2–4). Similarly, 30 VOCs showed significantly different levels in the resistant KPN in relation to the TSB medium, out of which 25 were released and five were taken up in bacteria culture.

**TABLE 2:** VOCs with the release profile directly related to the bacteria load.

Class	VOC	CAS	Strain	T0	T1	T2	T3	T4	T5	T6	Previously reported
**alcohols**	**Ethanol**	**64-17-5**	**Sens.**	1,71E+07	1,19E+07	9,39E+07	3,81E+08	4,42E+08	**4,63E+08**	**5,20E+08**	Storer et al., (2011); Rees et al., (2016)
			**Res.**	3,93E+07	2,33E+07	2,07E+08	**4,15E+08**	**4,74E+08**	**4,53E+08**	3,48E+08	
	**1-Propanol**	**71-23-8**	**Sens.**	1,46E+06	1,56E+06	2,28E+06	5,12E+06	**8,99E+06**	**1,98E+07**	***1,01E+08***	Boots et al. (2014)
			**Res.**	1,79E+06	1,16E+06	1,54E+06	3,15E+06	4,27E+06	5,07E+06	***1,41E+07***	
	**2-Butanol**	** 78-92-2**	**Sens.**	1,87E+06	1,98E+06	2,01E+06	2,11E+06	2,24E+06	2,34E+06	**6,71E+07**	Zechman et al. (1986)
			**Res.**	4,73E+05	4,13E+05	8,01E+05	7,35E+06	1,64E+07	1,44E+07	3,00E+05	
	**2-Methyl-1-Propanol**	**78-83-1**	**Sens.**	1,06E+06	7,97E+05	2,00E+06	6,34E+06	*1,83E+07*	***2,89E+07***	***1,05E+08***	Zechman et al., (1986); Lee et al., (1995); Kiviranta et al., (1998); Rees et al., (2017a)
			**Res.**	5,03E+05	5,46E+05	1,16E+06	3,40E+06	***8,01E+06***	***8,93E+06***	***2,52E+07***	
	**3-Methyl-1-Butanol**	**123-51-3**	**Sens.**	1,03E+06	1,39E+06	1,28E+07	4,93E+07	*1,63E+08*	***2,39E+08***	***6,49E+08***	Lee et al., (1995); Kiviranta et al., (1998); Jünger et al., (2012); Tait et al., (2014); Rees et al., (2017a)
			**Res.**	6,49E+05	3,53E+05	2,58E+06	1,30E+07	*6,41E+07*	***1,03E+08***	***4,58E+08***	
**aldehydes**	**Acetaldehyde**	**75-07-0**	**Sens.**	5,72E+06	3,90E+06	7,52E+06	1,43E+07	1,15E+07	**1,75E+07**	2,26E+07	
			**Res.**	1,19E+07	1,29E+07	1,47E+07	1,65E+07	1,72E+07	2,00E+07	2,26E+07	
	**Propanal**	**123-38-6**	**Sens.**	3,28E+05	4,22E+05	*3,50E+05*	3,48E+05	4,34E+05	7,71E+05	**3,76E+06**	
			**Res.**	2,91E+05	2,75E+05	*2,20E+05*	2,67E+05	2,67E+05	3,66E+05	**1,27E+06**	
	**Butanal**	**123-72-8**	**Sens.**	5,22E+05	7,30E+05	4,17E+05	5,11E+05	7,54E+05	7,72E+05	***2,67E+08***	
			**Res.**	4,12E+05	3,93E+05	2,07E+05	4,09E+05	6,34E+05	6,06E+05	***7,13E+07***	
**ketones**	**2,3-Butanedione**	**431-03-8**	**Sens.**	1,57E+06	1,46E+06	1,49E+06	7,03E+06	1,32E+07	*9,24E+06*	**1,70E+07**	Boots et al., (2014); Rees et al., (2016); Rees et al., (2017a)
			**Res.**	1,63E+06	8,18E+05	5,65E+05	9,69E+06	1,69E+07	*1,85E+07*	**3,79E+07**	
	**2-Butanone**	**78-93-3**	**Sens.**	1,51E+07	1,82E+07	1,78E+07	1,76E+07	1,74E+07	1,70E+07	**5,79E+08**	Zechman et al., (1986); Boots et al., (2014); Rees et al., (2017a)
			**Res.**	1,43E+07	1,25E+07	1,19E+07	1,26E+07	1,20E+07	1,16E+07	3,53E+08	
	**2-Pentanone**	**107-87-9**	**Sens.**	3,96E+05	5,46E+05	6,88E+05	6,68E+05	*8,09E+05*	***7,72E+05***	***2,49E+06***	Zechman et al., (1986); Rees et al.,(2016); Rees et al., (2017a)
			**Res.**	3,28E+05	4,17E+05	1,08E+06	**2,64E+06**	***2,76E+06***	***2,62E+06***	***7,33E+06***	
	**2-Heptanone**	**110-43-0**	**Sens.**	2,16E+05	*2,51E+05*	*2,34E+05*	2,67E+05	*2,70E+05*	*3,33E+05*	***8,83E+05***	Zechman et al., (1986); Lee et al., (1995); Rees et al.,(2016); Rees et al., (2017a); Rees et al., (2017b)
			**Res.**	7,31E+04	*9,67E+04*	*1,04E+06*	**3,48E+06**	***3,55E+06***	***3,20E+06***	***7,62E+06***	
	**2-Nonanone**	**821-55-6**	**Sens.**	2,83E+05	3,08E+05	*3,67E+05*	3,01E+05	*4,91E+05*	***5,54E+05***	***2,35E+06***	Zechman et al., (1986); Lee et al., (1995); Elgaali et al., (2002); Jünger et al., (2012); Rees et al., (2017a); Rees et al., (2017b)
			**Res.**	2,05E+05	6,48E+05	*7,96E+06*	**2,73E+07**	***2,47E+07***	***2,24E+07***	***3,00E+07***	
**esters**	**Methyl Acetate**	**79-20-9**	**Sens.**	1,82E+04	1,60E+04	1,59E+04	2,09E+04	2,03E+04	*3,56E+04*	**7,48E+04**	Rees et al. (2017a)
			**Res.**	2,88E+04	3,06E+04	3,35E+04	3,79E+04	4,36E+04	*5,52E+04*	7,48E+04	
	**Ethyl Acetate**	**141-78-6**	**Sens.**	3,10E+05	2,86E+05	7,48E+05	3,60E+06	9,19E+06	**1,47E+07**	**3,88E+07**	
			**Res.**	3,80E+05	1,65E+05	5,56E+05	4,18E+06	7,92E+06	**9,03E+06**	**1,72E+07**	
	**Ethyl Butyrate**	**105-54-4**	**Sens.**	n.d.	n.d.	n.d.	3,46E+04	7,21E+04	***1,32E+05***	**3,79E+05**	
			**Res.**	n.d.	n.d.	n.d.	3,06E+04	4,80E+04	*7,48E+04*	**1,97E+05**	
	**3-Methylbutyl Acetate**	**123-92-2**	**Sens.**	n.d.	n.d.	n.d.	4,56E+04	8,17E+04	*1,95E+05*	***2,23E+06***	
			**Res.**	n.d.	n.d.	n.d.	1,76E+04	5,25E+04	*8,66E+04*	***8,90E+05***	
**VSCs**	**Mercaptoacetone**	**24653-75-6**	**Sens.**	1,03E+05	9,27E+04	1,12E+05	3,57E+05	***5,99E+05***	***5,27E+05***	**5,66E+05**	
			**Res.**	9,02E+04	5,79E+04	7,15E+04	2,88E+05	*3,43E+05*	*3,54E+05*	**6,95E+05**	
**other**	**Ethyl Vinyl Ether**	**109-92-2**	**Sens.**	n.d.	n.d.	1,31E+04	2,06E+04	2,40E+04	***9,08E+04***	**2,08E+05**	
			**Res.**	n.d.	n.d.	n.d.	2,85E+04	2,99E+04	*2,50E+04*	9,24E+04	

Table legend: **Sens**., a sensitive strain of KPN; **Res**., a resistant strain of KPN; **bold value of peak area**, indicates the statistically significant difference (Kruskal-Wallis test) between the timepoint of interest and T0. *Underlined italic value of peak area*, indicates the statistically significant difference (U-Mann-Whitney test) between resistant and sensitive strain at the same timepoint.

The time points for headspace gas sampling were carefully chosen to cover the bacteria growth phase in detail and refer to the dynamic changes in emission of volatile metabolites to the kinetic of bacteria proliferation. In this regard, the following three time-dependent profiles of VOCs secretion were observed in this study.

#### Release proportional to bacteria load

**FIGURE 3:**
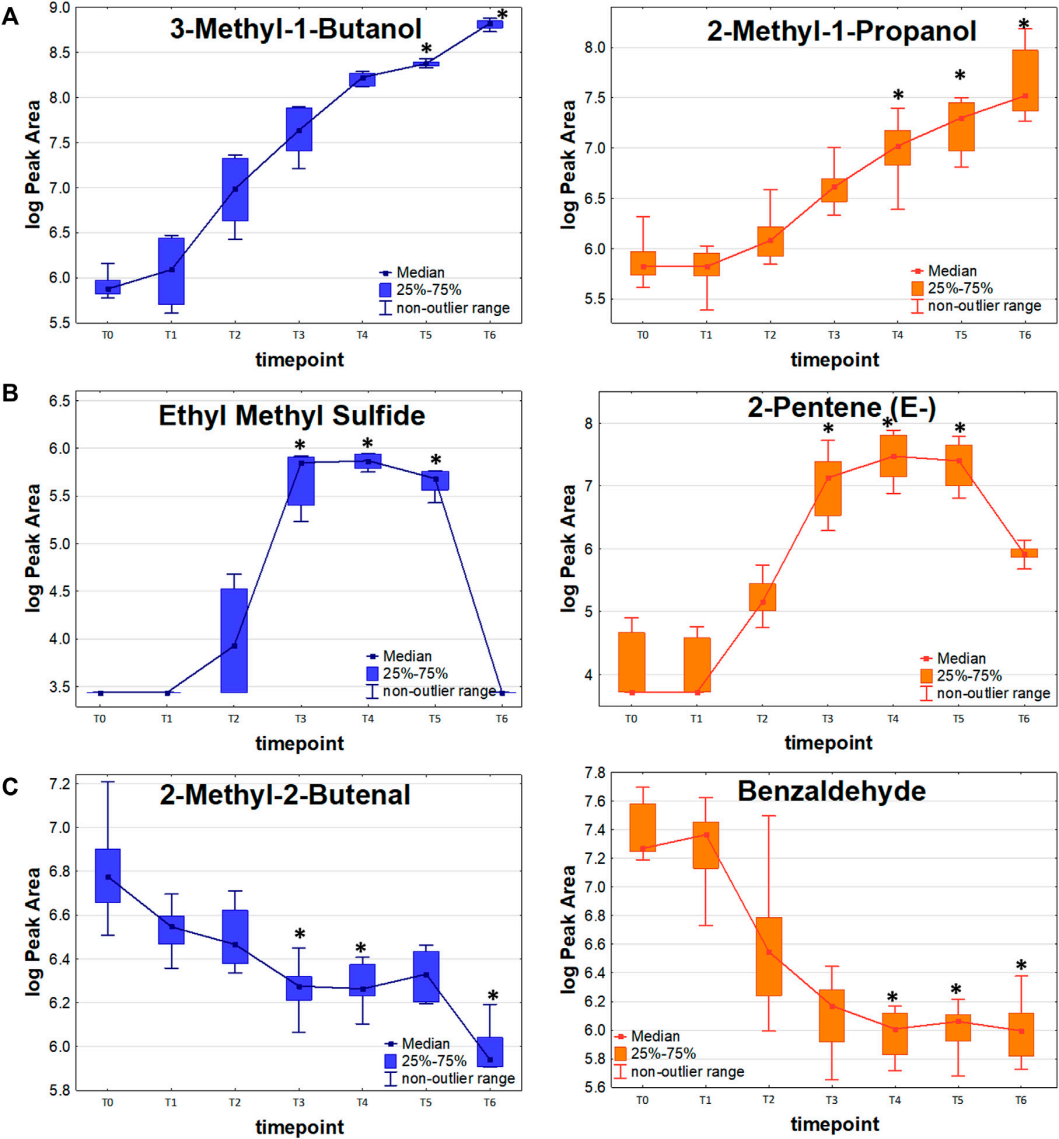
VOC release profiles in the function of bacteria proliferation. **(A)**: Release proportional to the bacteria load. **(B)**: Release with temporary maxiumum. **(C)**: Uptake of volatile metabolites by bacterial cells.

19 out of 44 significant VOCs observed for the sensitive KPN, and 14 out of 30 significant VOCs for the resistant KPN, were released in direct proportion to the bacteria load. In this case, the higher the absolute content of bacteria cells, the stronger the emission of volatile metabolites. The most prominent examples of this profile are alcohols, which all (except 1-butanol) exhibit this kinetic and reach a significantly higher abundance (in bacteria culture compared to medium control) during the first day of experiments at T3 (ethanol), T4 (1-Propanol, 2-methyl-1-propanol) or T5 (3-methyl-1-butanol). Alcohols exemplify the common observation in this study, namely a distinguishable profile already from the very early timepoints (T1/T2) followed by a clear increase (T2/T3/T4) but reaching the earliest statistical significance at relatively late phase (T4/T5) (compare Figure 3A and Table 2).

Further metabolites released by both strains during the first day of the experiment were mercaptoacetone and ethyl vinyl ether (first significance at T4 and T5, respectively) but both had relatively low abundance and substantial background level. Ketones constitute another important group of *K. pneumoniae* metabolites. 2,3-Butanedione and 2-butanone required a long bacteria proliferation to be found at sufficiently high levels in the headspace samples, whereby 2-pentanone, 2-heptanone and 2-nonanone were secreted in large quantities already after about 5 h since bacteria inoculation (T3). In turn, all esters and three aldehydes (acetaldehyde, propanal and butanal) were secreted in significant amounts by both strains at the earliest after 24 h of bacteria growth. Noticeably, the abundance of released VOCs was slightly higher for sensitive compared to resistant strains. Due to this reason, several compounds were found to be statistically significant solely for the sensitive KPN, for instance, acetaldehyde, 2-butanone, 2-butanol, methyl acetate and ethyl vinyl ether.

#### Temporary maximum

Amongst 44 VOCs relevant for the sensitive KPN, 13 reached the temporary maximum in their abundance either at the timepoint T3 (5 h 40min) or T4 (7 h 10min of incubation), after which a decline was observed. The analogous profile concerned 11 out of 30 VOCs significant for resistant strain (Figure 3B). The most prominent examples of this profile are volatile sulfur-containing compounds (VSCs) which all (except mercaptoacetone) undergo this kinetic and - after a temporary maximum - equalize their abundance in a final timepoint T6 roughly to the initial level before bacteria inoculation (T0) to the TSB medium. Also, the low-molecular hydrocarbons, including 2-methyl-2-butene, isoprene, (Z)-pentene and (E)-pentene, were released in the highest quantities at T4, after which their reduction in bacterial headspace was observed (Table 3). All compounds with this profile were released at slightly higher amounts by sensitive than by resistant strains of KPN. Some of them, like n-butyl acetate and ethyl n-octanoate, were observed at very low levels, close to the background, hence in particular cases, such as furan, the statistically significant difference between VOCs quantity in bacteria culture and reference medium could be found only for the sensitive strain.

**TABLE 3:** VOCs with the temporary maximum release profile.

Class	VOC	CAS	Strain	T0	T1	T2	T3	T4	T5	T6	Previously reported
**hydrocarbons**	**2-Methyl-2-Butene**	**513-35-9**	**Sens.**	1,14E+05	8,35E+04	2,13E+05	4,33E+06	***1,05E+07***	***7,91E+06***	1,04E+06	
			**Res.**	4,78E+04	6,18E+04	1,33E+05	**2,85E+06**	***3,57E+06***	***2,47E+06***	6,67E+05	
	**Isoprene**	**78-79-5**	**Sens.**	2,58E+05	2,97E+05	2,53E+06	**7,68E+06**	**6,08E+06**	3,14E+06	**7,16E+06**	Boots et al. (2014)
			**Res.**	2,11E+05	2,96E+05	2,78E+06	**6,31E+06**	**3,65E+06**	2,40E+06	2,33E+06	
	**2-Pentene (Z-)**	**627-20-3**	**Sens.**	5,78E+04	4,50E+04	3,41E+05	**2,53E+07**	***6,46E+07***	***4,71E+07***	1,03E+06	
			**Res.**	n.d.	n.d.	1,67E+05	**1,62E+07**	***1,85E+07***	***1,19E+07***	7,99E+05	
	**2-Pentene (E-)**	**627-20-3**	**Sens.**	6,00E+04	4,67E+04	3,43E+05	**2,57E+07**	***6,59E+07***	***4,75E+07***	1,00E+06	
			**Res.**	n.d.	n.d.	1,66E+05	**1,68E+07**	***1,91E+07***	***1,23E+07***	7,90E+05	
**alcohol**	**1-Butanol**	**71–363**	**Sens.**	1,06E+06	1,09E+06	1,54E+06	1,44E+07	**3,45E+07**	**3,91E+07**	*8,58E+06*	Rees et al. (2017a)
			**Res.**	4,80E+05	4,13E+05	8,01E+05	7,35E+06	**1,64E+07**	1,44E+07	*3,00E+05*	
**esters**	**n-Butyl Acetate**	**123-86-4**	**Sens.**	4,07E+04	4,03E+04	n.d.	8,19E+04	1,21E+05	***1,84E+05***	1,75E+05	Rees et al. (2017a)
			**Res.**	2,22E+04	1,22E+04	2,18E+04	3,46E+04	6,67E+04	***8,05E+04***	3,75E+04	
	**Ethyl n-Octanoate**	**106-32-1**	**Sens.**	n.d.	n.d.	1,14E+05	1,05E+06	**1,84E+06**	**1,30E+06**	*1,24E+06*	
			**Res.**	n.d.	n.d.	n.d.	1,34E+06	**1,40E+06**	**1,29E+06**	*3,36E+05*	
**sulfuric**	**Methanethiol**	**74-93-1**	**Sens.**	2,50E+05	3,18E+05	3,95E+05	**1,41E+06**	**1,70E+06**	8,73E+05	1,65E+06	Rees et al. (2017a)
			**Res.**	2,29E+05	1,93E+05	4,11E+05	**1,84E+06**	**1,94E+06**	1,21E+06	**1,26E+06**	
	**Carbon disulfide**	**75-15-0**	**Sens.**	4,25E+05	1,28E+06	1,16E+06	**1,26E+06**	**1,27E+06**	1,20E+06	6,03E+05	Rees et al. (2016)
			**Res.**	1,03E+06	1,13E+06	1,10E+06	1,08E+06	1,02E+06	9,03E+05	6,03E+05	
	**Dimethyl Sulfide**	**75-18-3**	**Sens.**	6,35E+05	9,82E+04	1,18E+06	**1,27E+07**	***1,60E+07***	*8,35E+06*	7,61E+06	Storer et al., (2011); Rees et al., (2016)
			**Res.**	3,68E+05	7,58E+04	1,19E+06	**9,99E+06**	***6,74E+06***	*4,17E+06*	7,22E+05	
	**Ethyl Methyl Sulfide**	**624-89-5**	**Sens.**	n.d.	n.d.	3,59E+04	**5,77E+05**	**1,39E+06**	**1,03E+06**	1,46E+05	
			**Res.**	n.d.	n.d.	1,05E+05	1,89E+06	**1,43E+06**	**6,50E+05**	n.d.	
	**Dimethyl sulfone**	**67-71-0**	**Sens.**	2,39E+05	7,71E+04	7,09E+05	1,58E+06	**1,88E+06**	**1,61E+06**	*5,07E+05*	
			**Res.**	4,32E+05	n.d.	4,45E+05	1,68E+06	**1,75E+06**	1,31E+06	*1,16E+06*	
**other**	**Furan**	**110-00-9**	**Sens.**	3,45E+05	3,64E+05	3,86E+05	4,49E+05	**5,38E+05**	***5,74E+05***	4,14E+05	Rees et al. (2017a)
			**Res.**	3,93E+05	2,70E+05	2,63E+05	2,88E+05	3,13E+05	*3,03E+05*	2,28E+05	

Table legend: **Sens**., a sensitive strain of KPN; **Res**., a resistant strain of KPN; **bold value of peak area**, indicates the statistically significant difference (Kruskal-Wallis test) between the timepoint of interest and T0. *Underlined italic value of peak area*, indicates the statistically significant difference (U-Mann-Whitney test) between resistant and sensitive strain at the same timepoint.

#### Uptake

Altogether, 12 VOCs were taken up by the sensitive strain and five compounds by the resistant strain of KPN (Table 4). Apart from 3-phenylfuran, heptane and three C4-hydrocarbons (1-butene, (Z)-2-butene, (E)-2-butene), the remaining VOCs inversely proportional to bacteria load were aldehydes (Figure 3C). Amongst them, 2-ethylacrolein, 2-methyl-2-butenal and benzaldehyde are the most efficiently used by growing bacteria, hence a significant decline in their amount in the culture could be observed already at T3.

**TABLE 4:** VOCs taken up by Klebsiella pneumonia in the course of the experiment.

Chemical class	VOC	CAS	Strain	T0	T1	T2	T3	T4	T5	T6	Previously reported
**hydrocarbons**	**(Z)-2-Butene**	**590-18-1**	**Sens.**	7,04E+05	3,57E+05	3,12E+05	**2,81E+05**	**1,00E+06**	3,41E+05	4,81E+05	
			**Res.**	6,40E+05	2,93E+05	2,26E+05	5,63E+05	2,30E+05	2,42E+05	2,40E+05	
	**(E)-2-Butene**	**624-64-6**	**Sens.**	1,89E+05	1,27E+05	*1,16E+05*	**7,49E+04**	**6,66E+04**	7,41E+04	**8,41E+04**	
			**Res.**	2,00E+05	1,04E+05	*6,40E+04*	5,11E+04	4,79E+04	4,74E+04	4,03E+04	
	**1-Butene**	**106-98-9**	**Sens.**	1,61E+05	1,10E+05	1,07E+05	**7,55E+04**	**6,79E+04**	**7,34E+04**	9,55E+04	
			**Res.**	1,70E+05	8,92E+04	7,48E+04	5,93E+04	5,97E+04	5,73E+04	5,62E+04	
	**Heptane (-n)**	** 142-82-5**	**Sens.**	1,23E+05	1,38E+05	1,02E+05	9,99E+04	1,31E+05	2,40E+05	**n.d.**	Rees et al. (2017a)
			**Res.**	1,31E+05	8,24E+04	6,76E+04	5,85E+04	5,59E+04	**6,35E+04**	**n.d.**	
**aldehydes**	**Methacrolein**	**78-85-3**	**Sens.**	9,82E+06	8,99E+06	9,19E+06	5,41E+06	8,66E+06	6,75E+06	**2,01E+06**	
			**Res.**	6,45E+06	5,90E+06	6,62E+06	6,42E+06	1,06E+07	8,07E+06	1,95E+06	
	**2-Ethylacrolein**	**922-63-4**	**Sens.**	4,40E+06	1,38E+06	1,22E+06	**4,64E+05**	**6,14E+05**	1,12E+06	***7,81E+05***	
			**Res.**	1,40E+06	7,82E+05	1,00E+06	7,44E+05	7,42E+05	8,53E+05	*2,08E+05*	
	**3-MethylButanal**	**590-86-3**	**Sens.**	1,34E+08	1,44E+08	6,49E+07	***3,23E+07***	*5,78E+07*	***2,96E+07***	*4,96E+07*	Jünger et al., (2012); Boots et al., (2014); Tait et al., (2014)
			**Res.**	1,04E+08	9,36E+07	7,99E+07	*1,59E+08*	*2,28E+08*	*2,11E+08*	*1,77E+08*	
	**2-Methyl-2-Butenal**	**1115-11-3**	**Sens.**	7,48E+06	3,57E+06	3,29E+06	**1,92E+06**	**1,93E+06**	2,19E+06	**1,03E+06**	
			**Res.**	3,20E+06	2,19E+06	2,49E+06	2,18E+06	2,10E+06	2,12E+06	**5,34E+05**	
	**Furfural**	** 98-01-1**	**Sens.**	7,49E+05	4,61E+05	*4,52E+05*	**2,64E+05**	**1,94E+05**	***2,13E+05***	*5,99E+05*	
			**Res.**	5,65E+05	3,23E+05	*2,21E+05*	1,89E+05	**1,11E+05**	***1,08E+05***	*1,71E+05*	
	**Hexanal**	**66-25-1**	**Sens.**	2,28E+05	2,41E+05	7,10E+04	7,12E+04	**8,93E+04**	**1,06E+05**	**3,38E+03**	Rees et al. (2017a)
			**Res.**	2,17E+05	1,29E+05	3,90E+04	6,81E+04	**2,66E+04**	**4,66E+04**	**4,25E+04**	
	**Benzaldehyde**	**100-52-7**	**Sens.**	3,19E+07	3,16E+07	*1,49E+07*	1,85E+06	**1,28E+06**	***1,30E+06***	*1,58E+06*	Boots et al. (2014)
			**Res.**	2,35E+07	1,51E+07	*2,03E+06*	2,72E+06	**8,31E+05**	***8,25E+05***	***7,44E+05***	
**other**	**3-Phenylfuran**	**13679-41-9**	**Sens.**	3,52E+06	3,08E+06	2,80E+06	2,58E+06	*2,63E+06*	*2,63E+06*	**1,56E+06**	
			**Res.**	3,05E+06	1,84E+06	1,75E+06	1,77E+06	*1,53E+06*	*1,45E+06*	9,66E+05	

Table legend: **Sens**., a sensitive strain of KPN; **Res**., a resistant strain of KPN; **bold value of peak area**, indicates the statistically significant difference (Kruskal-Wallis test) between the timepoint of interest and T0. *Underlined italic value of peak area*, indicates the statistically significant difference (U-Mann-Whitney test) between resistant and sensitive strain at the same timepoint.

### VOCs related to antimicrobial resistance

To reveal, whether volatile metabolites can be produced with significantly different profiles by resistant and sensitive strains of *K. pneumoniae*, the U-Mann-Whitney test was used for comparison of VOC quantities detected at the same timepoint of bacteria proliferation. Given the three profiles described in the previous section, only the timepoints corresponding to the maximum abundance of VOC are taken into account, this means T3 or T4 (temporary maximum profile), T5 and T6 (release proportional to bacteria load). Only these metabolites are considered, which were found at a significantly higher level in bacteria culture for at least one strain (either resistant or sensitive). Compounds present at the same level in bacteria and medium control and compounds taken up by proliferating microorganisms are not considered at all in this section.

Amongst compounds with the release profile directly proportional to the bacteria load, significantly higher levels in susceptible strains were observed for ethanol (at T6), 1-propanol (T4, T5, T6), 2-methyl-1-propanol (T5, T6), 3-methyl-1-butanol (T5, T6), propanal (T6), 2-butanone (T6), ethyl butyrate (T6), 3-methylbutyl acetate (T6), mercaptoacetone (T4, T5) and ethyl vinyl ether (T5, T6).

Similarly, metabolites with a temporary maximum in bacteria production that were found at significantly higher levels in the cultures of susceptible strains comprise 2-methyl-2-butene (T4, T5), isoprene (T4), (Z)-2-pentene (T4, T5), (E)-2-pentene (T4, T5), 1-butanol (T4, T5), n-butylacetate (T5), dimethylsulfide (T4) and furan (T4, T5).

**FIGURE 4:**
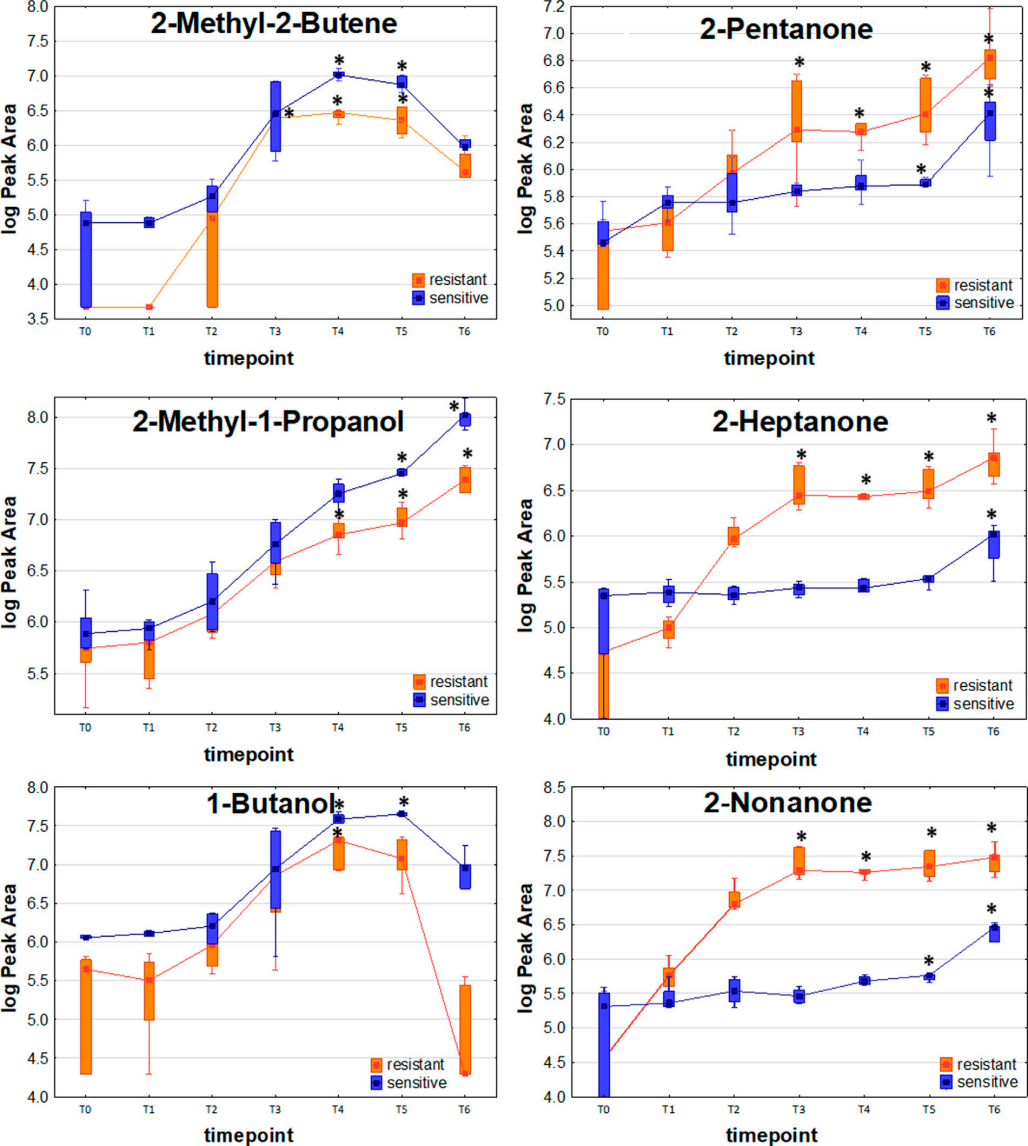
Comparison of exemplary VOC profiles released by sensitive and resistant strains of *Klebsiella pneumonia*.

The distinctive compounds were odd-carbon methyl ketones, which were the only VOCs found at significantly higher levels for the resistant *K. pneumoniae*, compared to the sensitive strain, including 2-pentanone (at T5, T6), 2-heptanone and 2-nonanone (both at T4, T5, T6). The comparison of release profiles for exemplary VOCs is given in Figure 4.

### Effect of antibiotics on VOCs release

To investigate the influence of carbapenem on VOCs emission from bacteria, 0.5 ml of imipenem solution in PBS with the concentration of 25 mg/100 ml was added to bacteria culture at T2 immediately after preconcentration of headspace gas on sorption tube (i.e. 4 h 50min after inoculation).

As a rule, antibiotics should trigger a far stronger effect on the susceptible strain than on the resistant one, which should also be true for the emission of volatile metabolites. Indeed, considering VOCs released by each strain separately (given in section 3.2), only two metabolites (isoprene and ethyl methyl sulfide) were found at significantly lower levels and only three metabolites (1-propanol, n-butyl acetate and hexanal) at significantly higher levels in resistant KPN after addition of imipenem. All other VOCs remained unaffected by the addition of antibiotics to the resistant KPN culture.

**FIGURE 5:**
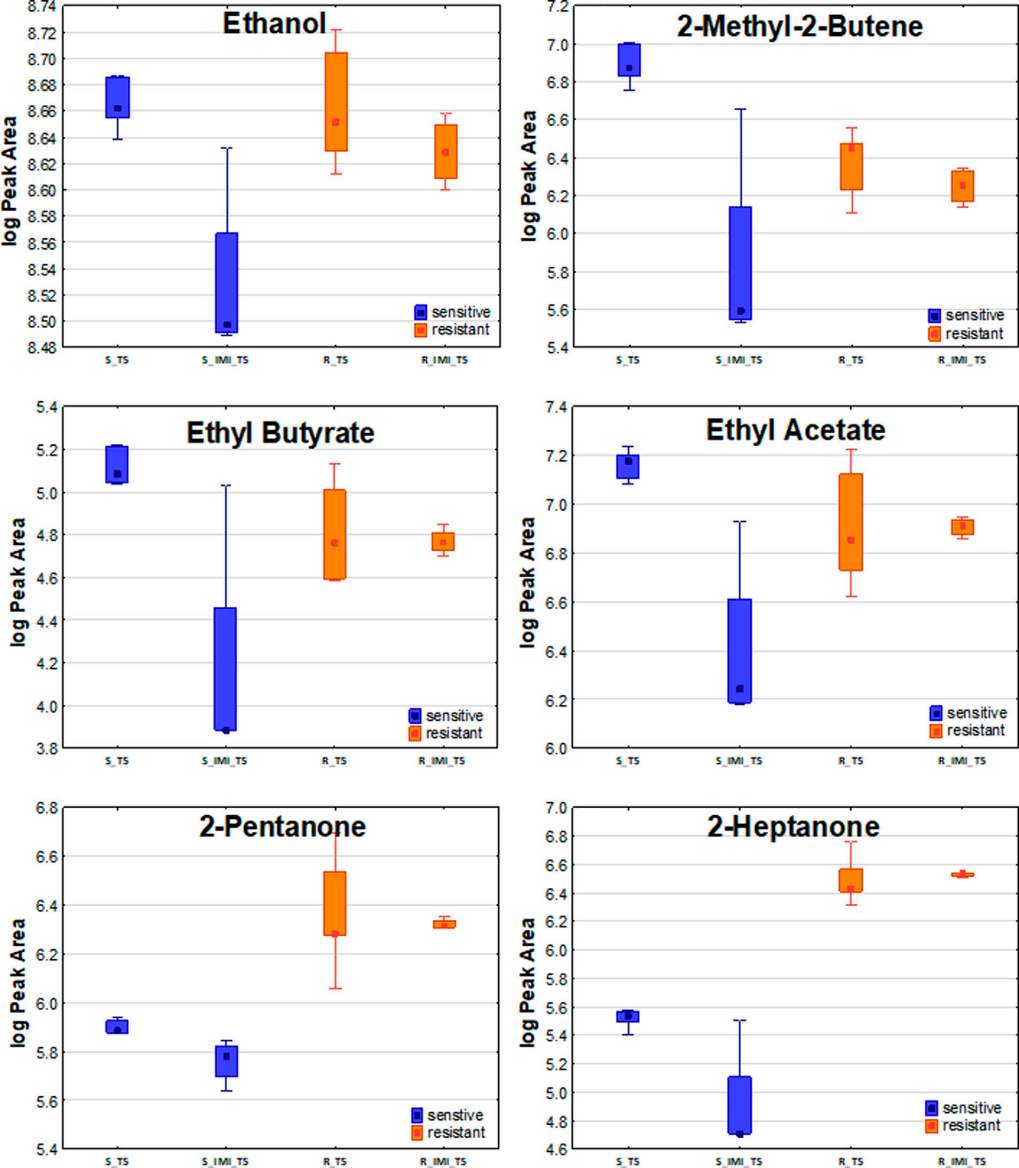
The effect of imipenem on metabolites secretion from Klebsiella pneumonia. A significant decline in VOCs amounts in bacterial headspace was found for sensitive strains (blue boxes on the left) but not for resistant ones (orange boxes on the right).

Concerning the susceptible strain, there was no single VOC that increased after the addition of imipenem. In turn, the abundance in bacterial headspace of 28 out of 44 VOCs significant for susceptible strains has declined after the addition of imipenem (Figure 5), and the profiles of the remaining 16 compounds were unchanged under these conditions. The complete list of compounds affected and unaffected by the addition of antibiotics to bacteria culture is given in Supplementary Table S1.

### VOCs released from clinical strains

**FIGURE 6:**
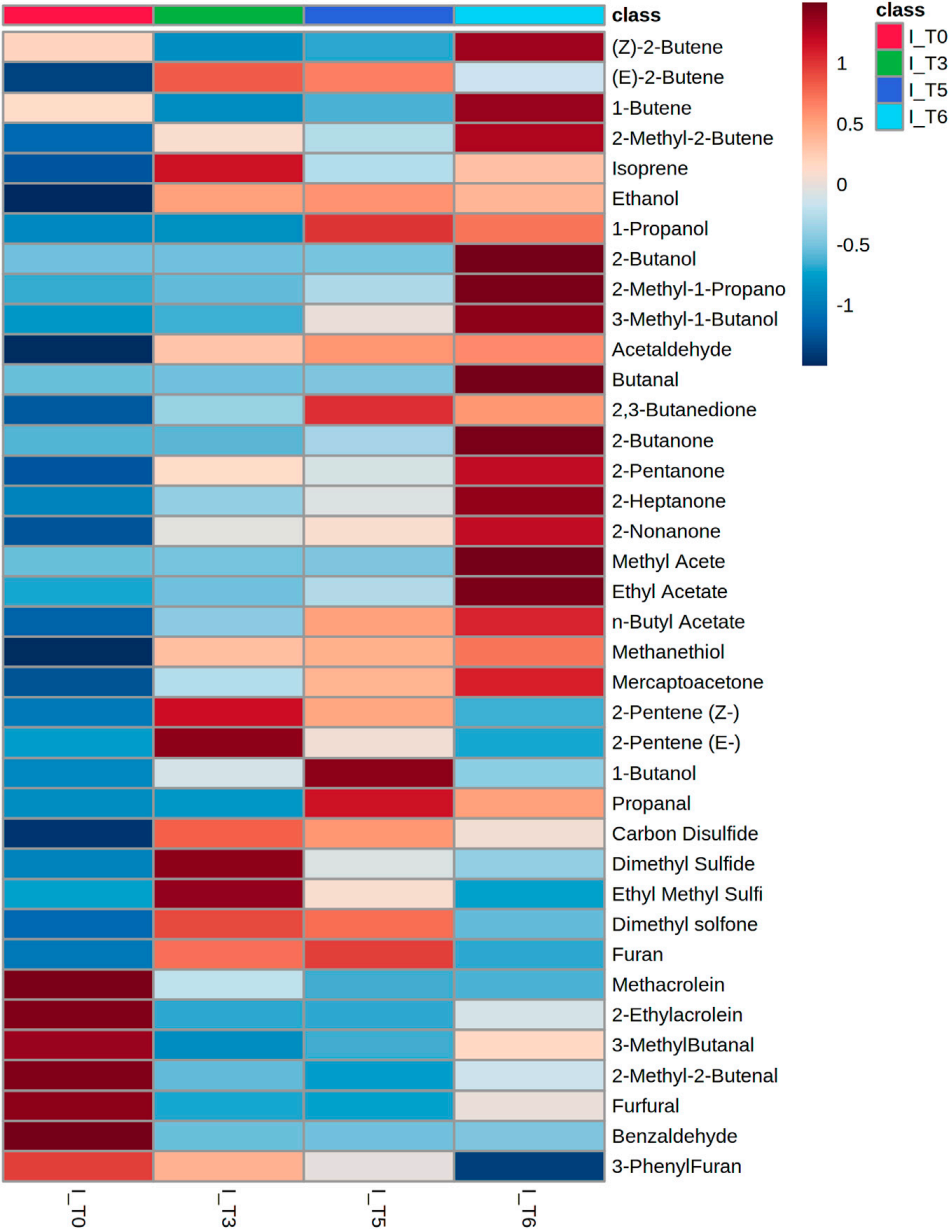
Heatmap representing the average abundance of all metabolites significantly released by clinical isolates of Klebsiella pneumonia (*n* = 19) at the respective timepoint of bacteria growth.

VOCs secretion profiles for 19 clinical strains isolated from BAL specimens collected from VAP patients were determined to compare the portfolio of metabolites released by reference and wild *K. pneumoniae*. Since only two isolates were imipenem-resistant, the entire set of clinical strains was referred to as the sensitive reference strain. Altogether 50 metabolites were found at significantly different levels in bacteria cultures compared to medium control and the aforementioned three profiles of VOCs production were observed (Supplementary Figure S1). Amongst them 31 were released proportionally to the absolute bacteria load, 11 exhibited the temporary maximum and another eight compounds were taken up by proliferating cells (Table 5). Noticeably, the vast majority of released VOCs have been found in all clinical isolates tested, thus they can be considered as a “core-volatilome” of wild *K. pneumonia* strains. The only exceptions from 100% occurrence are 2-methyl-2-butene (*n* = 18/19 at T3), 2-butanol (*n* = 18/19 at T5, *n* = 16/19 at T6), n-butyl acetate (*n* = 12/19 at T6), mercaptoacetone (*n* = 12/19 at T5 and *n* = 16/19 at T6), dimethyl sulfone (*n* = 18/19 at T6) and furan (*n* = 13/19 at T5). The overview of the VOCs abundance in clinical isolates at the chosen timepoints of bacteria growth is given in the form of a heatmap (Figure 6).

**TABLE 5:** Statistically significant VOCs observed in the culture headspace of KPN clinical isolates.

				T0		T3		T5		T6		
		VOC	CAS	Mean peak area	Occurrence (*n* = 19)	Mean peak area	Occurrence (*n* = 19)	Mean peak area	Occurrence (*n* = 19)	Mean peak area	Occurrence (*n* = 19)	Prev. Reported
RELEASE	**hydrocarbons**	**2-methyl-1-propene**	**115-11-7**	3,32E+06	19	**1,46E+06**	19	2,85E+06	19	9,93E+06	19	
		**(Z)-2-Butene**	**590-18-1**	6,37E+06	19	***9,07E+05***	19	**1,78E+06**	19	1,24E+07	19	
		**iso-Butane**	**75-28-5**	2,21E+06	16	1,04E+06	19	2,33E+06	19	**7,71E+06**	19	
		**(E)-2-Butene**	**624-64-6**	1,10E+06	19	***2,45E+07***	19	**2,27E+07**	19	*1,39E+07*	19	
		**1-Butene**	**106-98-9**	2,45E+05	19	***9,05E+04***	19	*1,30E+05*	19	4,38E+05	19	
		**2-Methyl-2-Butene**	**513-35-9**	2,91E+05	18	**6,38E+06**	18	***4,63E+06***	19	**1,22E+07**	19	
		**3-Methyl-1-Butene**	**563-45-1**	4,97E+05	19	3,76E+05	19	5,46E+05	19	**2,96E+06**	19	Rees et al. (2017a)
		**Isoprene**	**78-79-5**	2,77E+05	19	***7,34E+06***	19	**3,23E+06**	19	***4,88E+06***	19	Boots et al. (2014)
		**(E)-1,3-Pentadiene**	**2004-70-8**	8,10E+04	19	1,39E+05	19	1,40E+05	19	**1,89E+05**	19	
		**n-Nonane**	**111-84-2**	1,17E+05	19	1,54E+05	19	**4,53E+05**	19	**4,70E+05**	19	
	**alcohols**	**Ethanol**	**64-17-5**	1,23E+07	19	**5,03E+08**	19	***5,17E+08***	19	***4,71E+08***	19	Storer et al., (2011); Rees et al., (2016)
		**2-Propanol**	**67-63-0**	1,49E+07	19	**3,06E+07**	19	**2,70E+07**	19	**2,69E+07**	19	Rees et al. (2017a)
		**1-Propanol**	**71-23-08**	4,58E+05	19	**2,46E+06**	19	***6,99E+07***	19	***5,95E+07***	19	Boots et al. (2014)
		**2-Butanol**	** 78-92-2**	8,11E+05	8	6,95E+05	18	1,50E+06	15	***6,65E+07***	16	Zechman et al. (1986)
		**2-Methyl-1-Propanol**	**78-83-1**	3,90E+05	19	**3,72E+06**	19	***1,15E+07***	19	***6,17E+07***	19	Zechman et al., (1986); Lee et al., (1995); Kiviranta et al., (1998); Rees et al., (2017a)
		**3-Methyl-1-Butanol**	**123-51-3**	3,95E+05	19	**3,00E+07**	19	***1,60E+08***	19	***4,47E+08***	19	Lee et al., (1995); Kiviranta et al., (1998); Jünger et al., (2012); Tait et al., (2014); Rees et al., (2017a)
	**ald.**	**Acetaldehyde**	**75-07-0**	8,71E+06	19	**5,32E+07**	19	***5,99E+07***	19	**6,14E+07**	19	
		**Butanal**	**123-72-8**	6,51E+05	19	**2,58E+06**	19	**7,54E+06**	19	***2,95E+08***	19	
	**ketones**	**Methyl Vinyl Ketone**	**78-94-4**	2,25E+05	15	1,79E+05	19	2,91E+05	19	**4,65E+06**	19	
		**2,3-Butanedione**	**431-03-8**	1,53E+06	19	**1,62E+07**	19	**3,95E+07**	19	***3,16E+07***	19	Boots et al., (2014); Rees et al., (2016); Rees et al., (2017a)
		**2-Butanone**	**78-93-3**	1,33E+07	19	1,71E+07	19	**7,46E+07**	19	***4,79E+08***	19	Zechman et al., (1986); Boots et al., (2014); Rees et al., (2017a)
		**2-Pentanone**	**107-87-9**	4,17E+05	19	**1,68E+06**	19	***1,49E+06***	19	***2,69E+06***	19	Boots et al., (2014); Rees et al., (2016); Rees et al., (2017a)
		**2-Heptanone**	**110-43-0**	5,17E+04	19	2,51E+05	19	**3,61E+05**	19	***8,55E+05***	19	Zechman et al., (1986); Lee et al., (1995); Rees et al., (2016); Rees et al., (2017a); Rees et al., (2017b)
		**Acetophenone**	**98-86-2**	1,12E+06	17	1,44E+06	19	1,56E+06	19	**3,38E+06**	19	
		**2-Nonanone**	**821-55-6**	1,05E+04	19	1,34E+06	19	*1,49E+06*	19	***2,72E+06***	19	Zechman et al., (1986); Lee et al., (1995); Elgaali et al., (2002); Jünger et al., (2012); Rees et al., (2017a); Rees et al., (2017b)
	**esters**	**Methyl Acetate**	**79-20-9**	1,27E+04	19	1,80E+04	19	2,18E+04	19	***3,79E+05***	19	Rees et al. (2017a)
		**Ethyl Acetate**	**141-78-6**	3,79E+05	19	**7,33E+06**	19	***1,63E+07***	19	***7,99E+07***	19	
		**n-Butyl Acetate**	**123-86-4**	1,09E+04	4	9,52E+04	19	***1,95E+05***	19	**2,57E+05**	12	Rees et al. (2017a)
	**VSCs**	**Methanethiol**	**74-93-1**	1,57E+06	19	***8,56E+06***	19	**8,90E+06**	19	**1,00E+07**	19	Rees et al. (2017a)
		**Mercaptoacetone**	**24653-75-6**	3,67E+04	9	**1,99E+05**	12	***3,01E+05***	17	***4,11E+05***	16	
		**Dimethyl Disulfide**	**624-92-0**	1,57E+07	19	**4,97E+07**	19	**4,29E+07**	19	**7,47E+07**	19	Zechman et al., (1986); Storer et al., (2011); Rees et al., (2017a)
TEMPORARY MAXIMUM	**HC**	**(Z)-2-Pentene**	**627-20-3**	1,26E+05	19	***1,66E+07***	19	***1,15E+07***	19	**2,97E+06**	19	
		**(E)-2-Pentene**	**646-04-8**	5,69E+03	19	***5,31E+06***	19	*1,95E+06*	19	1,03E+05	19	
	**alc.**	**1-Butanol**	**71-36-3**	1,37E+05	19	**5,47E+06**	19	***1,56E+07***	19	3,46E+06	19	Rees et al. (2017a)
	**ald.**	**Propanal**	**123-38-6**	1,96E+05	19	3,20E+05	19	**4,51E+06**	19	**3,15E+06**	19	
		**2-Butenal**	**4170-30-3**	7,57E+05	1	**2,91E+06**	19	**5,90E+06**	19	3,47E+06	19	
	**ket.**	**Acetone**	**67-64-1**	1,00E+08	19	**1,68E+08**	19	**1,64E+08**	19	1,26E+08	19	Rees et al. (2017a)
	**sulfuric**	**Carbon disulfide**	**75-15-0**	3,11E+05	19	***8,09E+05***	19	**7,51E+05**	19	6,38E+05	19	Rees et al. (2016)
		**Dimethyl Sulfide**	**75-18-3**	3,14E+05	19	**1,67E+07**	19	**6,40E+06**	19	**4,14E+06**	19	Storer et al., (2011); Rees et al., (2016)
		**Ethyl Methyl Sulfide**	**624-89-5**	1,13E+03	1	***1,64E+06***	19	***6,42E+05***	19	0,00E+00	0	
		**Dimethyl sulfone**	**67-71-0**	1,36E+05	19	**1,92E+06**	19	***1,78E+06***	18	6,53E+05	18	
	**oth.**	**Furan**	**110-00-9**	3,47E+05	10	**1,22E+06**	13	***1,33E+06***	17	5,20E+05	19	Rees et al. (2017a)
UPTAKE	**aldehydes**	**Methacrolein**	**78-85-3**	7,51E+06	19	**3,03E+06**	19	**1,80E+06**	19	***1,88E+06***	19	
		**2-Methylpropanal**	**78-84-2**	9,15E+06	19	4,17E+06	19	**2,12E+06**	19	**1,53E+06**	19	
		**2-Ethylacrolein**	**922-63-4**	2,48E+06	19	***5,60E+05***	19	**5,62E+05**	19	***1,09E+06***	19	
		**3-Methylbutanal**	**590-86-3**	9,96E+07	15	***1,72E+07***	18	***2,50E+07***	18	5,50E+07	19	Jünger et al., (2012); Boots et al., (2014); Tait et al., (2014)
		**2-Methyl-2-Butenal**	**1115-11-3**	4,59E+06	19	***2,06E+06***	19	**1,82E+06**	19	***2,57E+06***	18	
		**Furfural**	** 98-01-1**	9,56E+05	18	***3,54E+05***	19	***3,47E+05***	19	**5,56E+05**	19	
		**Benzaldehyde**	**100-52-7**	2,12E+07	6	**1,18E+06**	19	***1,23E+06***	19	**1,64E+06**	15	
	**oth.**	**3-Phenylfuran**	**13679-41-9**	2,29E+06	18	1,99E+06	19	1,75E+06	19	***9,75E+05***	19	

HC, hydrocarbons; alc., alcohols; ald., aldehydes; ket., ketones; VSCs, volatile sulfur-containing compounds; oth, other compounds. **bold value of peak area**, indicates the statistically significant difference (Kruskal-Wallis test) between the timepoint of interest and T0. *Underlined italic value of peak area in blue font*,– indicates the statistically significant difference in the analogous experiment with reference strains of *K. pnuemoniae*.

Importantly, 38 out of 44 volatile metabolites significantly released by reference susceptible *K. pneumoniae* were also significant for clinical isolates. The remaining six compounds important only for reference strains (but not for clinical KPN) are heptane, hexanal, ethyl vinyl ether, 3-methylbutyl acetate, ethyl butyrate and ethyl n-octanoate. Most of these 38 compounds significant for reference and clinical strains belong to the core-volatilome present in all samples. The only exceptions in this group concern: 2-butanol, n-butyl acetate, mercaptoacetone, dimethyl sulfone and furan, occurrence of which is given in Table 5.

To verify whether a relationship between the biochemical activity of tested bacteria and their VOCs production can be found, the Phoenix panels (Becton Dickinson) were performed for all 19 clinical isolates. The only variables that differ among the 19 clinical strains were *1*) reduction of the resazurin-based index that indicates the use of citrate as a carbon source in biochemical processes, and *2*) capability for extended-spectrum beta-lactamase (ESBL) production. Therefore, only these two features were further used to divide all isolates into two nearly equal groups (positive: 1 and negative: 0), separately for citrate usage and ESBL presence. According to the non-parametric U-Mann Witheny test, there is no significant difference in the synthesis of the discussed VOCs between strains classified as ESBL+ and ESBL-, respectively citrate+ and citrate-.

## Discussion

Recently, culturing of microorganisms isolated from human material stands for a gold standard for identification of the pathogen causing nosocomial infections. Although this method is very specific, sensitive and provides information about antimicrobial resistance, it needs to be done by qualified personnel, requires invasive sampling of human specimens and results are known after up to 3 days, during which patient receives empirical therapy with broad-spectrum antibiotics. Therefore, alternative analytical approaches are searched for diagnosis of bacterial infections or at least supplementation of the conventional microbiological methods.

Several valuable clinical studies were pursued in the last decade to reveal the usefulness of breath analysis for the detection of bacteria presence in the respiratory tract or bloodstream of the critically ill. This approach assumes the qualitative and quantitative determination of bacterial volatile metabolites in exhaled breath gas of patients suspected of infection, whereby the mass spectrometric methods are often applied for this purpose due to their ability to the identification of an unknown substance. This strategy was used by Schnabel et al., in 2015 for the selection of 12 breath-VOCs correctly discriminating individuals with and without VAP (Schnabel et al., 2015). Another sound study was done in 2015 by Filipiak et al. who demonstrated that selected bacterial VOCs are not only present in the breath of VAP patients but also that concentrations of these metabolites change dynamically in the course of pneumonia and reflect the severity of infection (Filipiak et al., 2015). A further clinical study performed in 2016 by Gao et al. showed that breath analysis can be useful for differentiation of colonization in MV patients from actual bacterial infection causing VAP as well as an indication of the presence of causative pathogen (*Acinetobacter baumannii*) in the lower respiratory tract of the suspected patient (Gao et al., 2016). However, despite these evidences and the non-invasiveness of breath analysis, no volatile biomarker could reach the clinical relevance for reliable prediction of emerging bacterial infection or sepsis so far. This is related to study design which often relays on a very limited number of patients recruited from a single center accompanied by the lack of standardization in the applied analytical methodology and strategy for data interpretation. Altogether, it may result in random cohesions of investigated variables and incorrect conclusions (such as consideration of sample impurities as disease markers). For data interpretation, it is particularly important to correctly define the identity of the target metabolite, i.e. to confirm its bacterial origin in a model *in vitro* experiments with microorganisms, using reference standards for VOCs identification (for instance by GC-MS) in addition to only preliminary match of unknown spectra to MS library. Such an approach enables the elucidation of a biochemical pathway underlying bacterial VOCs production, increasing the unambiguity of the findings of clinical research. Therefore, the reference carbapenem-susceptible and -resistant strains of *Klebsiella pneumonia* were studied in this work under *in vitro* conditions to characterize the kinetics of VOCs release or uptake and to search for metabolites related to the antimicrobial resistance. The influence of antibiotic addition to bacteria culture on VOCs production was investigated in addition. The results gathered for reference strains were ultimately compared with the profiles of VOCs released from clinical strains of *K. pneumoniae* isolated from VAP patients.

### Bacteria proliferation

The clinical strains, depending on their actual origin, might be generally different in their basic metabolism properties. Moreover, they are usually subjected to many antimicrobials in a hospital environment, especially during infection, whereas reference strains were stored and cultured under well-controlled and unchanged conditions. Therefore, the growth kinetics of clinical strains might be of different types and explain statistically significant differences in the growth curves at particular timepoints. Thus, the patterns of VOCs released or synthesized by clinical and reference strains might be slightly different.

### Time-dependent profiles of metabolites production of *Klebsiella pneumoniae*

The dynamic changes in the kinetics of microbial VOCs production are related either to the total burden of bacterial cells or their metabolic activity. However, they may also imply clinical practice, as the metabolites released at the early stage of bacteria growth could indicate the emerging infection. In turn, compounds, secretion of which is directly proportional to the total bacteria load could be monitored in the course of infection, reflecting its progress or resolution (e.g. in the consequence of the applied antibiotic therapy).

#### Release proportional to the bacteria load

One of the most intriguing metabolites observed in our study were ketones, as all of them were released by bacteria with the same kinetics and their quantity was always directly proportional to the total bacteria load. The odd-carbon methyl ketones (2-pentanone, 2-heptanone and 2-nonanone) were significantly released already 5 h after inoculation of *K. pneumoniae* (T3), whereas 2-butanone and 2,3-butanedione required sufficiently higher bacteria density as their significant release was observed only at the last timepoint of the experiment (T6 = 24 h). All five ketones produced by *K. pneumonia*e in this study have also been reported in similar *in vitro* experiments by other researchers (Zechman et al., 1986; Jünger et al., 2012; Boots et al., 2014; Rees et al., 2016). 2-Butanone is formed through glucose metabolism in bacteria cells with 3-hydroxybutanone and 2,3-butanediol as the intermediate products undergoing final dehydration to ketones (Chen et al., 2015). 2,3-Butanedione is synthesized by bacterial decarboxylases from (2S)-2-hydroxy-2-methyl-3-oxobutanoic acid rising from pyruvate metabolism (Whiteson et al., 2014). 2-Pentanone, 2-heptanone and 2-nonanone originate most probably from the β-oxidation of fatty acids (Rees et al., 2018; Zhu et al., 2019)

The remaining compounds exhibiting this profile of secretion were alcohols (*n* = 5), esters (*n* = 4), aldehydes (*n* = 3), mercaptoacetone and ethyl vinyl ether. Amongst them, only alcohols (Zechman et al., 1986; Tait et al., 2014; Rees et al., 2016) and methyl acetate (Whiteson et al., 2014) were discussed as volatile metabolites of *K. pneumoniae* in other studies. Most probably, ethyl esters discussed in the here presented study originate from reactions of alcohols and acetyl-CoA generated *via* fatty acids metabolism (Saerens et al., 2010).

Intriguing profiles were observed for aldehydes, whereby only low molecular and very volatile acetaldehyde, propanal and butanal were released by both reference KPN strains of reaching the statistical significance at the last timepoint of the experiment (T6). All other aldehydes (containing four and more carbon atoms in the molecule) were only taken up by *K. pneumonia*.

#### Temporary maximum

In the second profile of VOC emission from bacteria, the maximum abundance of metabolites falls to the timepoints T3 and T4, which correspond to the second half of the logarithmic phase of *K. pneumoniae* growth under the applied conditions. It indicates that dynamics of crucial cellular processes such as the division of bacteria cells and related with that the highest metabolic activity play a major role in the production of VOCs with this profile, whereas an absolute bacteria density does not affect VOCs secretion at all.

Whereas all hydrocarbons exhibiting this profile were low-molecular and very volatile (2-methyl-2-butene, both 2-pentene isomers, isoprene), the esters in this group were considerably larger molecules, such as n-butyl acetate and ethyl n-octanoate. Important observation concerns the volatile sulfur-containing compounds (VSCs), as they all (except mercaptoacetone) exhibit the same release profile with a temporary maximum. Interesting is the case of ethyl methyl sulfide, which was never reported before by other researchers in similar *in vitro* studies with pathogenic bacteria. However, further studies are needed to elucidate whether it may be used as the specific volatile marker of *K. pneumoniae* differentiating it from other bacteria species. The pathway underlying the synthesis of ethyl methyl sulfide is probably the degradation of sulfur-containing amino acids such as methionine and cysteine (Andersson et al., 2004). Dimethyl disulfide (DMDS) and carbon disulfide (CS2), which exhibit a similar profile for sensitive and resistant strains, are formed through transformations of dimethylsulfoniopropionate (Reisch et al., 2011) and as a result of the degradation of amino acids containing sulfur. One of the most universal metabolites synthesized by numerous bacteria species is methanethiol, which was also observed in this study with a temporary maximum profile. *Klebsiella pneumoniae* is capable to synthesize this metabolite *via* the transsulfurylation pathway of amino acids containing sulfur atoms (Seiflein and Lawrence, 2006).

Metabolites exhibiting the release profile with a temporary maximum reach high abundances already at the relatively early phase of bacteria growth and therefore deserve attention in further studies as potential markers for early detection of the emerging bacterial infection.

#### Uptake

An important observation in this study is a significant uptake of aldehydes, which start with the logarithmic phase of bacteria growth. Several aldehydes, such as methacrolein, 2-methyl-2-butenal and benzaldehyde serve as an energy source for proliferating bacteria. For instance, benzaldehyde is reduced by benzaldehyde dehydrogenase to yield NADPH as alternative energy in *Pseudomonas putida* (Zahniser et al., 2017). Apparently, a similar process also takes place in *Klebsiella pneumoniae*. Aldehydes are also the intermediate products in the alcohol synthesis (Gu et al., 2017), what explains their significant decline in the course of the experiment with a simultaneous exponential release of alcohols.

### VOCs related to antimicrobial resistance

One of the most striking observations in our study was the significantly stronger release of the odd-carbon methyl ketones such as 2-pentanone, 2-heptanone and 2-nonanone from carbapenem-resistant than susceptible strains. These ketones originate from β-oxidation of fatty acids in bacteria cells. Wen et al. described the metabolome of carbapenem-resistant *K. pneumoniae* infection in plasma they obtained similar results which showed that patients with this infection had altered fatty acid metabolism (Wen et al., 2021). These results are a good predictor of antibiotic resistance markers but require further research.

### Effect of antibiotics on VOCs release

It has been previously shown that antibiotics have an impact on the metabolome of bacteria. Imipenem is a beta-lactam that inhibits cell wall synthesis (Dwyer et al., 2014; Balsalobre et al., 2019). In this regard, a clear change in VOC profiles after addition of imipenem to the bacteria culture manifested in our study in the significantly reduced VOC levels what apparently results from killing of the susceptible cells. Although dead bacteria may secrete to the environment numerous substances from the intracellular matrix, there was no single volatile metabolite (in the molecular range covered in our experiment) that could significantly increase after addition of carbapenem to the susceptible *K. pneumoniae*.

In the carbapenem-resistant strain, the response to imipenem affected other VOCs than in the susceptible strain. Similar results were obtained by Foschi et al. (Foschi et al., 2018). The following compounds: (E)-2-butene, 2-butanone, 3-phenylfuran (all three released), acetaldehyde and carbon disulfide (both taken up) were found in our experiments at significantly different level at T5 in resistant strain after addition of imipenem compared to the analogous T5 in the culture of resistant KPN but without this antibiotic. Noticeably, these five VOCs were not metabolized by normally growing resistant K. pneumoniae, as no significant difference could be ever seen at any timepoint compared to the medium control (T0). Another compounds metabolized by normally growing resistant KPN had significantly altered profile after antibiotic treatment. These are n-butyl actetate, 1-propanol, isoprene, ethyl methyl sulfide (all released) and hexanal (taken up). Particularly interesting profile was observed for 1-propanol and 2-butanone as their release from resistant KPN was significantly stronger after imipenem addition. The reason remains unknown, but it seems that mechanisms responsible for antimicrobial resistance (for instance carbapenemases) which are activated under stressed conditions (such as addition of imipenem) may be somehow involved in the metabolic pathways underlying VOCs production. Alternatively, a bacterial quorum sensing may be activated after antibiotic addition and trigger the synthesis of signaling metabolites. Nevertheless, these suggestions require further detailed investigations, which exceeds by far a scope of the here presented study.

Overall, the rapid reduction in the levels of numerous volatile metabolites reflects the eradication of **susceptible** bacteria due to imipenem treatment, hence may have potential to be used as an alternative approach for therapeutic drug monitoring in a non-invasive manner by breath gas analysis.

### VOCs released from clinical strains

To elucidate whether metabolites produced by reference strains under *in vitro* conditions are relevant also for clinical strains causing VAP in ICU patients, the profiles of VOC release were investigated for 19 isolates of *K. pneumonia* collected from BAL samples. Noticeably, a very high inter-strain stability in VOCs release was observed for clinical isolates, since as many as 44 out of 50 significant metabolites were found with 100% occurrence in clinical samples and stands for a “core-volatilome” of clinical KPN strains. Moreover, the majority of them (33 out of 44) were confirmed in the model *in vitro* experiments with reference *K. pneumoniae*, where their dependence on antibiotic treatment and relation to antimicrobial resistance were investigated. The here obtained results provide a good prognostic for further studies on alternative markers of pulmonary infections in VAP patients. Different kinetics of bacterial VOCs production may improve the versatility of an ultimate breath test. In this regard, compounds exhibiting temporary maximum, which are highly abundant for fast-proliferating cells (despite their relatively low cell density) may be very useful for the early detection of an emerging infection. On the other hand, metabolites released directly proportional to the absolute bacteria load may reflect the severity of the infection and therefore be used for monitoring disease resolution in response to antibiotic therapy.

Based on the previous studies the following VOCs have been identified as unique for *K. pneumoniae*: 1,3-butadiene, butyraldehyde, longifolene, octyl acetate, tridecanol, dodecenal, (E)-2-hexyl ester, butanoic acid, and 5,5-dodecadinyl-1 12-diol (Karami et al., 2017). Interestingly, none of these compounds have been detected in a group of the strains included in our study. Therefore, further studies are necessary to establish a detailed pattern of compounds to apply them as biomarkers of a particular pathogen presence.

Meanwhile, as it has been confirmed, differentiation of bacteria causing *pneumonia* is possible when appropriate incubation conditions are applied, also without the necessity of microbiological testing of bacteria culture. Therefore, VOC-based tests have the potential to improve the identification of relevant pathogens and their differentiation (Kunze-Szikszay et al., 2021).

## Study limitations

One of the study limitations is a low number of carbapenem-resistant strains among clinical isolates. More studies are needed to examine the impact of carbapenem on VOC levels in strains isolated from the patients.

## Conclusion

This study provides the insight into kinetics of VOCs release from *Klebsiella pneumoniae* under normal conditions and under the influence of imipenem addition into both, susceptible and resistant strains. There different profiles were observed for VOCs metabolism: *1*) release proportional to the bacteria load, *2*) release with a temporary maximum at the highest proliferation activity and *3*) uptake as a material or energy source. The odd-carbon methyl ketones: 2-pentanone, 2-heptanone, 2-nonanone were released at significantly higher levels from the resistant *K.pneumoniae* compared to the susceptible strain, which suggest the possibility of using them as antimicrobial resistance markers. In the culture of susceptible strain treated with imipenem, a rapid reduction in the levels of numerous volatile metabolites was observed in the consequence of bacteria eradication.

As it has been previously demonstrated, the identification of a chosen microorganism is possible based on a measurement of selected volatiles in the culture headspace. In the nearest future, it may have great potential to be applied in medical microbiology laboratories as a potential diagnostic tool. The idea of great relevance would be to translate these observations into a breath analysis approach to arrive at a point-of-care applicable non-invasive diagnosis of bacterial infections. Further model *in vitro* experiments with bacteria cultures are needed to provide the basis for future multicenter clinical studies verifying the relevance of selected volatile biomarkers in clinical practice.Our results indicate that analysis of bacterial VOCs may have potential to be used as an alternative approach for detection of emerging VAP and for therapeutic drug monitoring. Such a far-reaching aim as the non-invasive diagnosing of pneumonia with distinguishing the causing pathogen, would certainly require a set of metabolites assigned to the bacteria of interest instead of a single compound, that ultimately has to be validated in the multicenter clinical studies enrolling well controlled cohorts of MV patients. Our *in vitro* experiments provide the basis for this approach indicating differences and similarities in VOCs production kinetics from carbapenem-resistant and susceptible *Klebsiella pneumoniae* under normal and stressed conditions (imipenem addition). Further experiments with clinically relevant pathogens are needed as a model *in vitro* studies to reveal which metabolites are the best candidates to arrive at a point-of-care applicable non-invasive diagnosis of bacterial infection by breath gas analysis.

## Data Availability

The datasets presented in this article are not readily available because they contribute to the larger project and may be available by request to the corresponding author only after its accomplishment. Requests to access the datasets should be directed to wojciech.filipiak@cm.umk.pl.
