# Supplementary material for: GC-MS profiling of volatile metabolites produced by Klebsiella pneumoniae
Source: Front Mol Biosci. 2022 Oct 18;9:1019290. doi: 10.3389/fmolb.2022.1019290 (PMC9623108; doi:10.3389/fmolb.2022.1019290)
Supplement: Supplementary file 1 [file Table1.docx]

**SUPPLEMENTARY TABLE 1:** Statistical comparison of VOC profiles after addition of imipenem (IMI) to the sensitive and resistant *Klebsiella pneumonia* cultures. The p-values were calculated with U-Mann-Whitney non-parametric test (with FDR correction) for VOC level at timepoint T5 (8h 40min of bacteria incubation) without and after imipenem addition, separately for sensitive and resistant strain. See table legends for more details.

| **Compound** | **original profile** | **„p” sensitive** | **after IMI addition** | **„p” resistant** | **after IMI addition** |
| --- | --- | --- | --- | --- | --- |
| Acetaldehyde | S ↑ | 0,014215 | ↓ | 0,006928 | ↓ Res. N.S. |
| Methanethiol | S ↑, R ↑ | 0,337356 | **no change** | 0,142681 | **no change** |
| (Z)-2-Butene | S ↓ | 0,014215 | ↓ | 0,699676 | **no change** |
| (E)-2-Butene | S ↓ | 0,069955 | **no change** | 0,006928 | ↑ res. N.S. |
| 1-Butene | S ↓ | 0,069955 | **no change** | 0,938503 | **no change** |
| Ethanol | S ↑, R ↑ | 0,014215 | ↓ | 0,396066 | **no change** |
| Furan | S ↑ | 0,014215 | ↓ | 0,589155 | **no change** |
| Propanal | S ↑, R ↑ | 0,337356 | **no change** | 0,053758 | **no change** |
| Carbon Disulfide | S ↑ | 0,025182 | ↓ | 0,025261 | ↓ Res. N.S. |
| Dimethyl Sulfide | S ↑, R ↑ | 0,014215 | ↓ | 0,699676 | **no change** |
| 2-Methyl-2-Butene | S ↑, R ↑ | 0,014215 | ↓ | 0,315875 | **no change** |
| Methyl Acete | S ↑ | 0,014215 | ↓ | 0,244552 | **no change** |
| Ethyl Vinyl Ether | S ↑ | 0,014215 | ↓ | 0,938163 | **no change** |
| Isoprene | S ↑, R ↑ | 0,014215 | ↓ | 0,037243 | ↓ |
| 2-Pentene (Z-) | S ↑, R ↑ | 0,025182 | ↓ | 0,938503 | **no change** |
| 2-Pentene (E-) | S ↑, R ↑ | 0,025182 | ↓ | 0,699676 | **no change** |
| 1-Propanol | S ↑, R ↑ | 0,014215 | ↓ | 0,006928 | ↑ |
| Methacrolein | S ↓ | 0,014215 | ↓ | 0,589155 | **no change** |
| 2,3-Butanedione | S ↑, R ↑ | 0,014215 | ↓ | 0,396066 | **no change** |
| Butanal | S ↑, R ↑ | 0,240956 | **no change** | 0,816961 | **no change** |
| 2-Butanone | S ↑ | 0,014215 | ↓ | 0,016771 | ↑ res. N.S. |
| Ethyl Methyl Sulfide | S ↑, R ↑ | 0,014215 | ↓ | 0,037243 | ↓ |
| Ethyl Acetate | S ↑, R ↑ | 0,014215 | ↓ | 0,487454 | **no change** |
| 2-Butanol | S ↑ | 0,014215 | ↓ | 0,589155 | **no change** |
| 2-Methyl-1-Propanol | S ↑, R ↑ | 0,014215 | ↓ | 0,938503 | **no change** |
| 1-Butanol | S ↑, R ↑ | 0,069955 | **no change** | 0,075983 | **no change** |
| 2-Ethylacrolein | S ↓ | 0,069955 | **no change** | 0,589155 | **no change** |
| Mercaptoacetone | S ↑, R ↑ | 0,014215 | ↓ | 0,053758 | **no change** |
| 3-MethylButanal | S ↓ | 0,337356 | **no change** | 0,142681 | **no change** |
| 2-Pentanone | S ↑, R ↑ | 0,042826 | ↓ | 0,699676 | **no change** |
| 2-Methyl-2-Butenal | S ↓ R ↓ | 0,069955 | **no change** | 0,589155 | **no change** |
| 3-Methyl-1-Butanol | S ↑, R ↑ | 0,025182 | ↓ | 0,142681 | **no change** |
| Furfural | S ↓ R ↓ | 0,042826 | ↓ | 0,938503 | **no change** |
| Hexanal | S ↓ R ↓ | 0,226342 | **no change** | 0,034660 | ↑ |
| Ethyl Butyrate | S ↑, R ↑ | 0,013062 | ↓ | 0,938503 | **no change** |
| n-Butyl Acetate | S ↑, R ↑ | 0,069955 | **no change** | 0,025261 | ↑ |
| Dimethyl sulfone | S ↑, R ↑ | 0,109820 | **no change** | 0,938503 | **no change** |
| 3-Methylbutyl Acetate | S ↑, R ↑ | 0,040325 | ↓ | 0,699676 | **no change** |
| 2-Heptanone | S ↑, R ↑ | 0,040325 | ↓ | 0,396066 | **no change** |
| Benzaldehyde | S ↓ R ↓ | 0,915106 | **no change** | 0,315875 | **no change** |
| 2-Nonanone | S ↑, R ↑ | 0,042826 | ↓ | 0,142681 | **no change** |
| 3-PhenylFuran | S ↓ | 0,749119 | **no change** | 0,006928 | ↑ res. N.S. |
| Ethyl n-Octanoate | S ↑, R ↑ | 0,069955 | **no change** | 0,142681 | **no change** |

Table legend: S = sensitive strain, R = resistant strain, ↑= increase of VOC amount after imipenem addition, ↓ = decrease of VOC amount after imipenem addition, res. N.S. = compound was not significant for pure resistant KPN strain
