# Supplementary figures and images for: GC-MS profiling of volatile metabolites produced by Klebsiella pneumoniae
Source: Front Mol Biosci. 2022 Oct 18;9:1019290. doi: 10.3389/fmolb.2022.1019290 (PMC9623108; doi:10.3389/fmolb.2022.1019290)

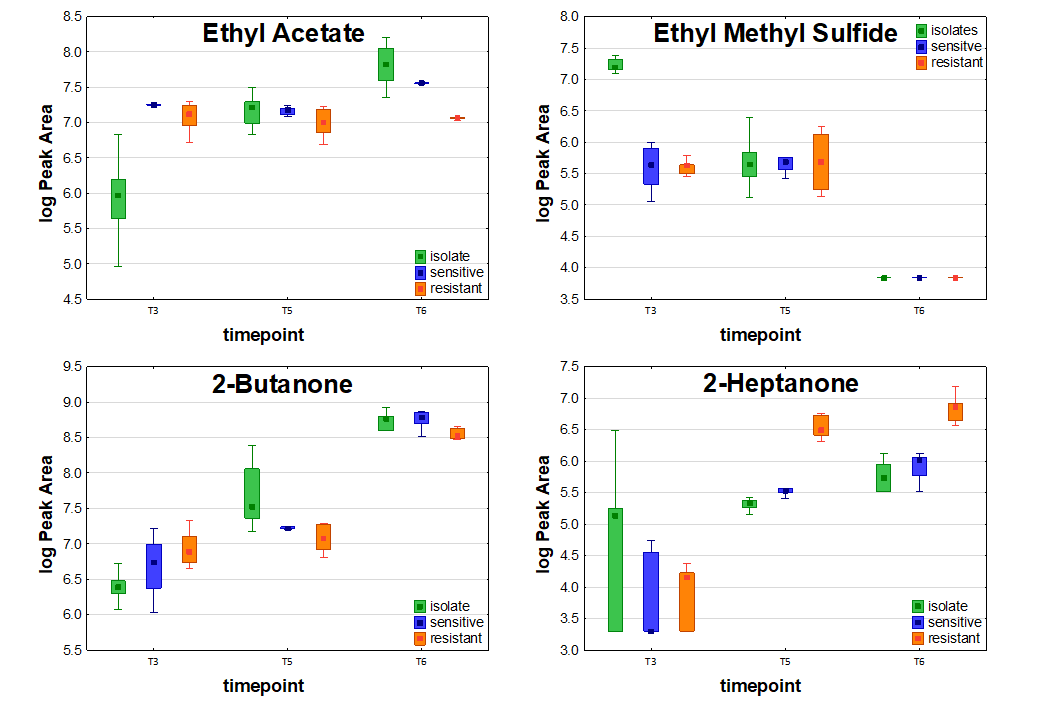

Supplement: Supplementary file 2 [file Image1.TIF]
